# Similar dose-dependence of motor neuron cell death caused by wild type human TDP-43 and mutants with ALS-associated amino acid substitutions

**DOI:** 10.1186/1423-0127-20-33

**Published:** 2013-05-30

**Authors:** Lien-Szu Wu, Wei-Cheng Cheng, Che-Kun James Shen

**Affiliations:** 1 Institute of Molecular Medicine, National Taiwan University, Taipei, Taiwan; 2 Institute of Molecular Biology, Academia Sinica, Nankang, Taipei, 115, Taiwan

**Keywords:** TDP-43, ALS Mutations, Protein stability, Spinal motor neuron cells, Apoptosis

## Abstract

**Background:**

TDP-43, a multi-functional DNA/ RNA-binding protein encoded by the *TARDBP* gene, has emerged as a major patho-signature factor of the ubiquitinated intracellular inclusions (UBIs) in the diseased cells of a range of neurodegenerative diseases. Mutations in at least 9 different genes including *TARDBP* have been identified in ALS with TDP-43 (+)-UBIs. Thus far, the pathogenic role(s) of the more than 30 ALS-associated mutations in the *TARDBP* gene has not been well defined.

**Results:**

By transient DNA transfection studies, we show that exogenously expressed human TDP-43 (hTDP-43), either wild type (WT) or 2 different ALS mutant (MT) forms, could cause significantly higher apoptotic death rate of a mouse spinal motor neuron-like cell line (NSC34) than other types of cells, e.g. mouse neuronal Neuro2a and human fibroblast HEK293T cells. Furthermore, at the same plasmid DNA dose(s) used for transfection, the percentages of NSC34 cell death caused by the 2 exogenously expressed hTDP-43 mutants are all higher than that caused by the WT hTDP-43. Significantly, the above observations are correlated with higher steady-state levels of the mutant hTDP-43 proteins as well as their stabilities than the WT.

**Conclusions:**

Based on these data and previous transgenic TDP-43 studies in animals or cell cultures, we suggest that one major common consequence of the different ALS-associated TDP-43 mutations is the stabilization of the hTDP-43 polypeptide. The resulting elevation of the steady state level of hTDP-43 in combination with the relatively low tolerance of the spinal motor neurons to the increased amount of hTDP-43 lead to the neurodegeneration and pathogenesis of ALS, and of diseases with TDP-43 proteinopathies in general.

## Background

The TAR-DNA-binding protein 43 (TDP-43)-encoding gene, *TARDBP*, is well conserved among the multicellular organisms from *C. elegans* to human [1,2]. Of the multiple isoforms encoded by the *TARDBP* gene, the 43 kDa TDP-43 protein is the most abundant one expressed in all tissues [3,4], mainly in the nucleus but some also residing in the cytoplasm [4,5]. TDP-43 appears to be a general transcription repressor [3,5,6], a splicing factor [7,8], and a neuronal activity-responsive factor [4]. Not surprisingly, intact *TARDBP* gene is indispensible for normal early development of the mouse embryos [9-12]. Lately, TDP-43 has emerged as the major patho-signature protein of the ubiquitinated intracellular inclusions (UBIs) in the diseased brain/ neuron cells of a range of neurodegenerative diseases, two major ones being the frontotemporal lobar degeneration with ubiquitin-positive, tau- and α-synuclein -negative inclusions (FTLD-U) and amyotrophic lateral sclerosis (ALS) [13-15]. Biochemical analyses have revealed that human TDP-43 (hTDP-43) is promiscuously modified/ processed in the affected regions of the brains and spinal cords of the FTLD-U and ALS patients, respectively [13-15]. Loss-of-function of TDP-43 as well as gain-of-cytotoxicity, as the result of the promiscuous modifications of TDP-43, have been suggested to lead to the pathogenesis of FTLD-U and as ALS with the TDP-43(+) UBIs [6,15-18] and references therein].

The molecular and cellular basis for the pathogenesis of either ALS or FTLD-U is poorly understood yet. Mutations in 11 different genes, including the long studied superoxide dismutase 1 (SOD 1) and *TARDBP*, have been identified to be associated with 10% of ALS [19], which is a disease with age-dependent degeneration of the spinal cord motor neurons [20]. Furthermore, the majority of the ALS cases, including those the disease genes of which have not been identified yet, are signalized with the TDP-43(+)-UBIs [21]. Interestingly, more than 30 different ALS-associated *TARDBP* mis-sense substitutions have been identified, almost all of which are mapped in the glycine-rich domain of TDP-43 [15,16,22]. A number of DNA transfection/ microinjection experiments in cell cultures or cell lines have been carried out to analyze the cyto-toxicities of different ALS-associated hTDP-43 mutants in comparison to the wild type [23-27]. For instance, Q331K and M337V accelerate spontaneous hTDP-43 aggregation in yeast cells [23]. On the other hand, while both the wild type hTDP-43 and 3 mutant forms of hTDP-43 (A315T, G348C, and A382T) induce death of primary motor neurons but not cells from Neuro2a and COS cell lines, the mutant forms are more potent than the wild type hTDP-43 in the induction of neuron death [24]. hTDP-43^A315T^ is also more toxic to the primary rat cortical neurons than the wild type hTDP-43 [25]. Furthermore, hTDP-43^Q331K^ and TDP-43^M337V^ induce oxidative injury of the motor neuron-like NSC34 cells [26]. One unanswered question from these studies is why, in general, the ALS-associated mutants of hTDP-43 are more cyto-toxic than the wild type hTDP-43. Notably, in most, if not all, of the above mentioned cell culture and cell line studies, the relative cellular levels of the exogenous proteins were not quantified and compared between the wild type and mutant hTDP-43.

In the following, we show that two randomly chosen ALS mis-sense mutations of the *TARDBP* gene both increase the stability of the TDP-43 polypeptide in motor neuron-like cells as well as in non-motor neuron cells. In addition, the mutant hTDP-43 polypeptides as well as the wild type hTDP-43 induce significant apoptosis of motor neuron-like cells, but much less so in non-motor neuron cells, in a dose-dependent manner. Thus, the major role of the ALS-associated hTDP-43 mutations appears to be the enhancement of the steady-state level of hTDP-43 through stabilization of the polypeptide in the spinal motor neurons, which have a low tolerance to the elevated cellular level of TDP-43 in comparison to the non-motor neuron cells.

## Methods

### Construction of expression plasmids

Wild type (WT) human hTDP-43 with addition of a Myc epitope tag to its 3’-end was generated by PCR of human brain cDNA using the following primers: forward, 5'-CCG CTC GAG CGG ATG TCT GAA TAT ATT CGG GTA AC -3'; reverse, 5'-TCT AGA GCT ACA GAT CCT CTT CCG AGA TGA GTT TTT GTT CCA TTC CCC AGC CAG AAG AC-3'. The A315T and N390D mutations were introduced into the WT cDNA by site-directed mutagenesis using the QuikChange® Site-Directed Mutagenesis Kit (Strategene). The three hTDP-43 cDNAs were first cloned into the pGEM-T vector (Promega, Madison, WI). Following sequence confirmation, the cDNA inserts were subcloned into the XhoI/ XbaI sites of a pEF vector. Experimental research that is reported in the manuscript was performed with the approval of an appropriate ethics committee.

### Antibodies

The commercial antibodies used in this study included a rabbit anti-TDP-43 polyclonal antibody (pAb) raised against a.a 1–260 of human TDP-43 and recognizing human as well as mouse TDP-43 (Gene Tex), a human specific mouse monoclonal antibody (mAb) against the same TDP-43 sequence (2E2-D3) (Abnova), anti-Myc mAb (LTK), anti-α-tubulin (Sigma), anti-cleaved caspase 3 (Asp175) (Ac-cap3) (Cell Signaling), and anti-Hsp70 (Chmicon).

### Cell cultures and DNA transfection

NSC34 cells were maintained in DMEM (Invitrogen) supplemented with 10% FBS (Invitrogen) and 1% antibiotics (100 IU/mL penicillin and 100 g/mL streptomycin). Neuro2a cells were maintained in MEM (Invitrogen) supplemented with 10% FBS (Invitrogen), 1% antibiotics, and 1% sodium pyruvate (Invitrogen). SHSY5Y cells were maintained in DMEM/F12 (Invitrogen) with 10% FBS (Invitrogen), 1% antibiotics, and 1% sodium pyruvate (Invitrogen). The cells were transfected with the empty pEF vector and the expression plasmids, respectively, using Lipofectamine 2000 transfection reagent (Invitrogen) following the manufacturer’s protocol. The amount of the plasmid DNA used in each transfection was kept at 20 μg/10^6^ cells by supplement with the pEF vector DNA. After transfection for different hrs, the cells were harvested and analyzed by Western blotting. In general, under the conditions used by us, the transfection efficiencies of NSC34, Neuro2A, SHSY5Y, and HEK293 were approximately 50%, 70%, 70%, and 90%, respectively.

### Cell death assay with use of Caspase-Glo 3/7

After incubation with the transfectants, the cells were split and seeded with two different densities, 2 × 10^3^ cells/well and 8 × 10^3^ cells/well, and allowed to grow for 24 hr and 72 hr, respectively. On the average, the cells were at 30% confluency before the assay. For the assay, Caspase-Glo 3/7 reagent (Promega) was added to all the wells in a 1:1 ratio following the manufacturer's instructions. Cells with addition of 5 μM staurosporine (0.1% final DMSO; Sigma) for 6 hr were used as a positive control [28]. After shaking at room temperature for 30 min, the lysates were analyzed with an Microplate Reader (Vector). A total of 3 replicates were performed. To determine the fold changes of caspase 3/ 7 activities, four independent experiments were carried out. The data were expressed as means ± SD. The differences in the caspase 3/ 7 activities among the variants were assessed by the ANOVA test. An unpaired two-tailed Student’s *t*-test was then used to obtain the *p* values associated with comparisons between the MT and WT.

### Cell death assay by immunofluorescence staining

The cells were fixed in 4% paraformaldehyde in phosphate-buffered saline (PBS), permeabilized with 0.1% Triton X-100 (Sigma) in PBS for 5 min, blocked with 10% donkey serum in PBS for 2 hr, and incubated overnight at 4°C with the primary antibodies anti-hTDP-43 (2E2-D3), anti-Myc, and anti-cleaved caspase 3, respectively . The primary antibodies were visualized with secondary antibodies conjugated with Alexa Fluor 488 or Alexa Fluor 561 (Molecular Probe), and the nuclei were detected using DAPI (4,6-diamino-2-phenylindole). The patterns of immunofluorescence staining were analyzed in a LSM710 confocal microscope (Zeiss). For quantification of cleaved caspase-3 positive cells, several random fields/ sample were analyzed and the percentages of transfected cells displaying anti-cleaved caspase 3 staining signals and apoptotic nuclei were calculated (N=150 cells, duplicate in one experiment). To determine the percentages of dead cells, four independent experiments were carried out. The data were expressed as means ± SD. The differences in % of the Ac-cap 3-positive cells among the variants were assessed by the ANOVA test. An unpaired two-tailed Student’s *t*-test was then used to obtain the *p* values associated with comparisons between the MT and WT.

### Western blot analysis

Cells were lysed in RIPA buffer (0.1% SDS, 1% Nonidet P-40, 0.5% sodium deoxycholate, 5mM EDTA, 150mM NaCl, 50mM Tris-HCl, pH 8.0) supplemented with protease inhibitors (Roche) and phosphatase inhibitors (Sigma). The protein concentrations of the lysates were measured using the Bio-Rad protein assay reagent on a Beckman Coulter DU-800 machine. The lysates were then resolved by SDS–PAGE and immunoblotted with the indicated antibodies. Quantification of the immunoblot band intensities was performed with use of the Image J software (NIH). There independent experiments on each cell line were carried out for densitometry analysis. The data were expressed as means ± SD. The differences in the relative levels of hTDP-43 among the variants were assessed by the ANOVA test. An unpaired two-tailed Student’s *t*-test was then used to obtain the *p* values associated with comparisons between the MT and WT.

### Protein degradation analysis

Cells were transfected with different expression plasmids encoding Myc-tagged versions of WT hTDP-43 and the two MT hTDP-43. Cycloheximide (50 μg/ mL; Sigma) was added to the media at 40 hr post-transfection. At various time points thereafter, the transfected cells were lysed and the amounts of the Myc tagged TDP-43 proteins were measured by Western blot analysis using the anti-hTDP-43 antibody (2E2D3) or anti-Myc antibody (LTK). Four independent experiments on each cell line were carried out for densitometry analysis. The data were expressed as means ± SD. The differences in relative levels of hTDP-43 among the variants were assessed by the ANOVA test. An unpaired two-tailed Student’s *t*-test was then used to obtain the *p* values associated with comparisons between the MT and WT.

### Statistical analysis

The data obtained from independent experiments are expressed as the mean ± S.D.. The differences among the variants were assessed by the ANOVA test. An unpaired two-tailed Student’s t-test was then used to obtain the *p* values associated with comparisons between the MT and WT.

## Results and discussion

### Overexpression of Wild Type (WT) or Mutant (MT) Human TDP-43 (hTDP-43) induced apoptotic death of motor neuron cells

**Figure 1:**
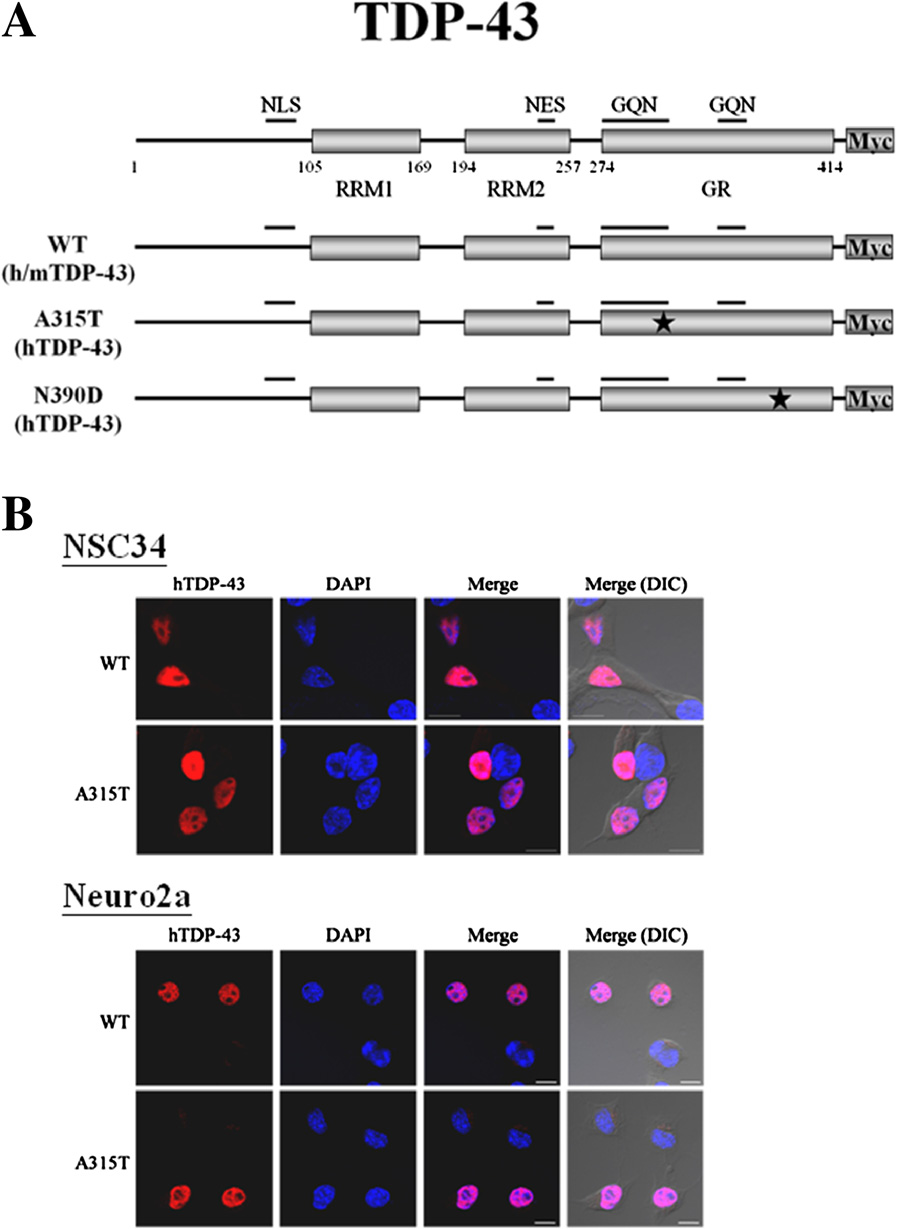
**Immunofluorescence staining analysis of NSC34 and Neuro2a cells overexpressing WT and MT hTDP-43. (A)** Physical maps showing the domains of human TDP-43 (hTDP-43) and mouse TDP-43 (mTDP-43). NLS, nuclear localization signal; NES, nuclear export signal; GR, glycine-rich sequence; GQN, glycine (G), glutamine (Q) and/ or asparagine (N)-rich sequence. For hTDP-43, the cDNA inserts in the 3 expression plasmids encoding the WT and the two mutant forms of hTDP-43 are shown. The positions of the nucleotide substitutions are indicated by the stars. **(B)** Representative photographs showing the immunofluorescence staining patterns of transfected cells. NSC34 (upper two rows) and Neuro2a (lower two rows) cells were transfected with plasmids expressing the WT hTDP-43 and hTDP-43^A315T^, respectively, and analyzed by immunofluorescence staining at 48 hr post-transfection with use of the 2E2D3 antibody recognizing hTDP-43 and DAPI staining the nucleus. Note the predominant nuclear localization of the exogenous WT and MT hTDP-43 in both types of cells. Scale bar, 10 μm.

To examine the relative cytotoxicities of WT and MT hTDP-43 in motor neuronal cells and non-motor neuronal cells, respectively, plasmids encoding the WT hTDP-43 and hTDP-43 carrying two different ALS-associated mutations (A315T and N390D) (Figure 1A) were transiently transfected into mouse NSC34, mouse Neuro2a, human HEK293T, and human SHSY5Y cells. Of these cell lines, NSC34 was established by fusion of the embryonic mouse spinal cord cells, which were enriched in the motor neurons, with mouse neuroblastoma cells [29]. It exhibited a number of motor neuron properties and was used widely as a cellular model system for motor neuron studies [30]. On the other hand, the differentiated neuron-like Neuro2a was established from a spontaneous neuronal tumor of a strain A albino mouse [31]. SHSY5Y cells was an human neuroblastoma cell line [32]. HEK 293T was an embryonic human kidney fibroblast cell line [33]. The two ALS-associated hTDP-43 mutations were chosen because of their identifications in more than one patient and by more than one group [15,16]. As seen in Figure 1B, the sub-cellular localization of the exogenous hTDP-43 was mainly nuclear in both transfected NSC34 and Neuro2a cells. Similar patterns were observed for transfected HEK 293T and SHSY5Y cells (data not shown). Consistent with the previous studies [24,34], the WT or MT hTDP-43 could be seen to form cytosolic aggregates in only a few percent of the transfected cells (data not shown).

**Figure 2:**
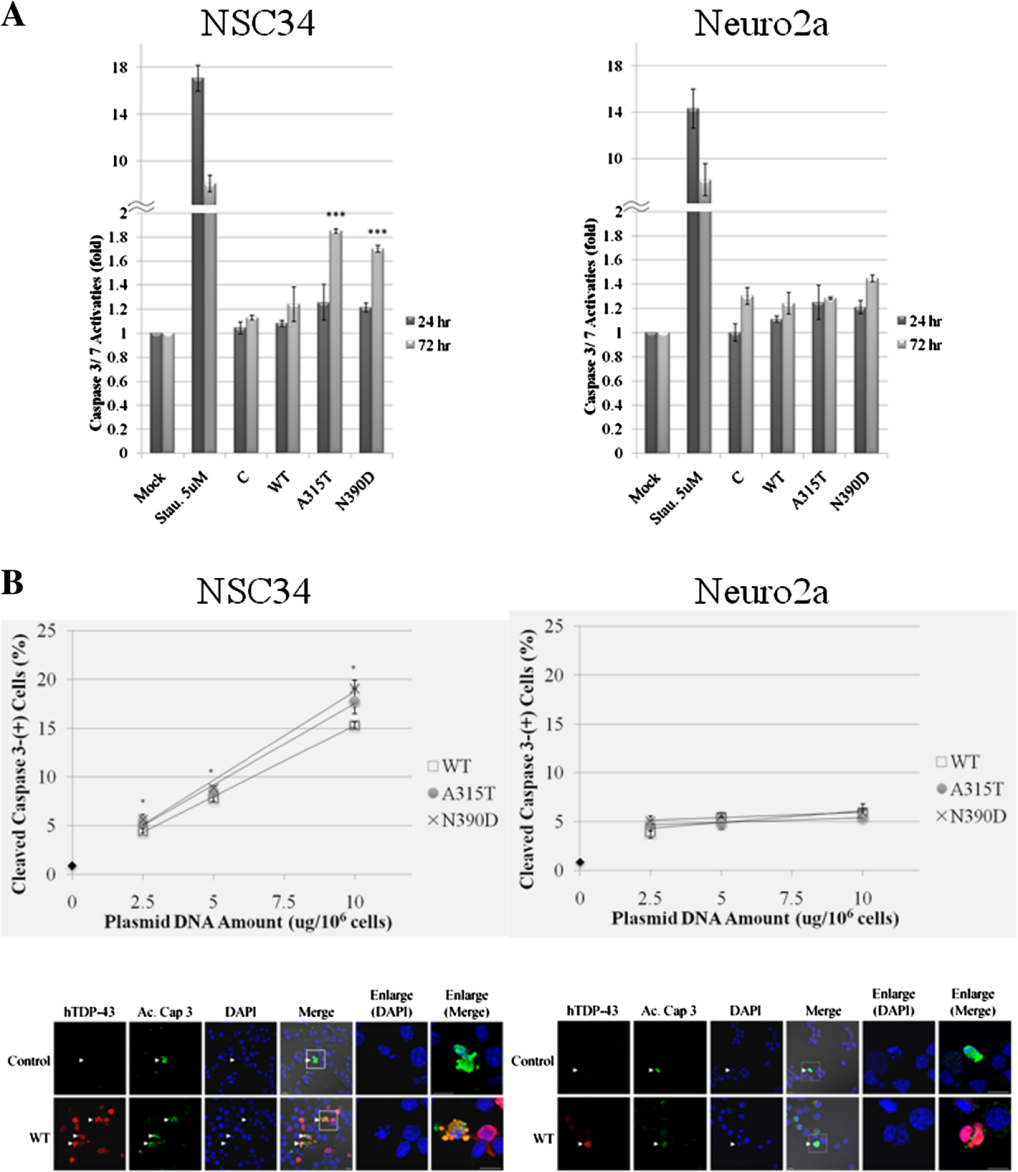
**Apoptotic assays of transfected NSC34 and Neuro2a cells. (A)** Comparison of apoptotic death induced by exogenous WT and MT hTDP-43. Cell death was assayed by measurement of the activities of caspase 3/ 7 at 24 hr and 72 hr post-transfection with the expression plasmids (5 ug/ 10^6^ cells). Mock, cells without transfection; C, cells transfected with the pEF vector; Stau. 5 uM, cells treated with 5 uM of staurosporine for 6 hr to induce apoptosis. The folds of the caspase activities relative to that of the Mock sample were calculated and shown. Note the significant increase of the caspase 3/ 7 activities in NSC34 cells (left panel), but not Neuro2a cells (right panel), induced by the two MT hTDP-43 forms at 72 hr post-transfection. **(B)** Comparison of plasmid dose-dependent apoptotic death induced by exogenous WT and MT hTDP-43. Apoptotic cell death was determined by immunofluorescence staining of cleaved caspase 3. The extents of apoptotic cell death of NSC34 cells and Neuro2a cells at 72 hr post-transfection with different amounts of the expression plasmids were assayed by immunofluorescence staining with the antibodies 2E2D3 and Ac-cap 3. Means of three independent experiments (S.D.) are plotted in the upper 2 panels, with the % of hTDP-43-positive cells that are also Ac-cap 3-positive as a function of the doses of transfection. Approximately 1% of cells transfected with the pEF vector were Ac-cap3 positive (◆ in the two plots). Representative photographs are shown below the plots, with the apoptotic nuclei/ cleaved caspase 3-positive cells indicated by the arrowheads. For both the NSC34 and Neuro2a sets, two Ac-cap 3-positive cells (the boxed areas) are magnified for better visualization. Scale bar, 10 μm. *, *p<*0.05; **, *p<*0.01; ***, *p<*0.001.

To examine the apoptotic cell death induced by overexpression of the WT hTDP-43 in comparison to the MT hTDP-43, we measured activities of the effector apoptotic caspases 3 and 7 of NSC34 (Figure 2A, left panel), Neuro2a (Figure 2A, right panel), and HEK 293T cells (data not shown) using a luminescent assay [35]. As seen, transfection of 10^6^ cells with 5 μg of plasmids overexpressing WT hTDP-43 (Figure 2A) or wild type mouse TDP-43 (mTDP-43) (Additional file 1: Figure S1) had little effect on the caspase 3/ 7 activities. On the other hand, NSC34 cells overexpressing the MT hTDP-43 at 72 hr, but not 24 hr, post-transfection exhibited 45-70% increases of the activities of caspase 3/ 7 in comparison to the vector control (left panel, Figure 2A). In contrast, the caspase 3/ 7 activities of Neuro2a cells overexpressing the MT hTDP-43 increased by only little (right panel, Figure 2A). Result of transfected HEK 293T cells was similar to Neuro2a (data not shown). Taken together, at the same dose (5 μg/ 10^6^ cells) of the expression plasmid(s) used for transfection, overexpression of the two MT hTDP-43 forms caused a motor neuronal cell type-specific cytotoxicity significantly higher than that by the WT hTDP-43.

### Motor neuronal cell-specific apoptotic death induced by both MT and WT hTDP-43 were dose-dependent

To examine whether the neurotoxicity of NSC34 cells caused by the overexpressed MT hTDP-43 was dose-dependent, we transfected NSC34 and Neuro2a cells with different amounts of the individual expression plasmids. The extents of apoptotic cell death were then analyzed by immunostaining with anti-cleaved caspase 3. Interestingly, MT hTDP-43 as well as WT hTDP-43 caused apoptotic cell death of the NSC34 cells in a dose-dependent manner, with the proportion of cleaved caspase 3-positive cells increased from 4-6% at the dose of 2.5 μg plasmid/ 10^6^ cells to 15-19% at 10 μg plasmid/ 10^6^ cells (upper left panel, Figure 2B). Furthermore, at each dose used for transfection, both MT hTDP-43 forms showed higher toxicities than the WT. Also, the differences of the effects between MT and WT hTDP-43 increased as higher amounts of the expression plasmids were used for transfection (upper left panel, Figure 2B). Similar to Figure 2A, overexpression of either MT or WT form of hTDP-43 caused much smaller increase (~4%) of the cell mortality of Neuro2a (upper right panel, Figure 2B) or HEK 293 cells (data not shown). Interestingly, overexpression of the wild type mTDP-43 also caused the selective neuronal apoptosis of the NSC34 cells in comparison to Neuro2a cells (Additional file 2: Figure S2). These data suggested that overexpression of either WT or MT hTDP-43 could cause significantly higher cytotoxicity in the motor neuronal-type cells than non-motor neuron cells. For some reason, however, the MT hTDP-43 appeared to be more toxic to NSC34 cells than the WT hTDP-43 or WT mTDP-43.

### ALS-associated hTDP-43 mutations stabilized hTDP-43 in NSC34 as well as in Neuro2a Cells

**Figure 3:**
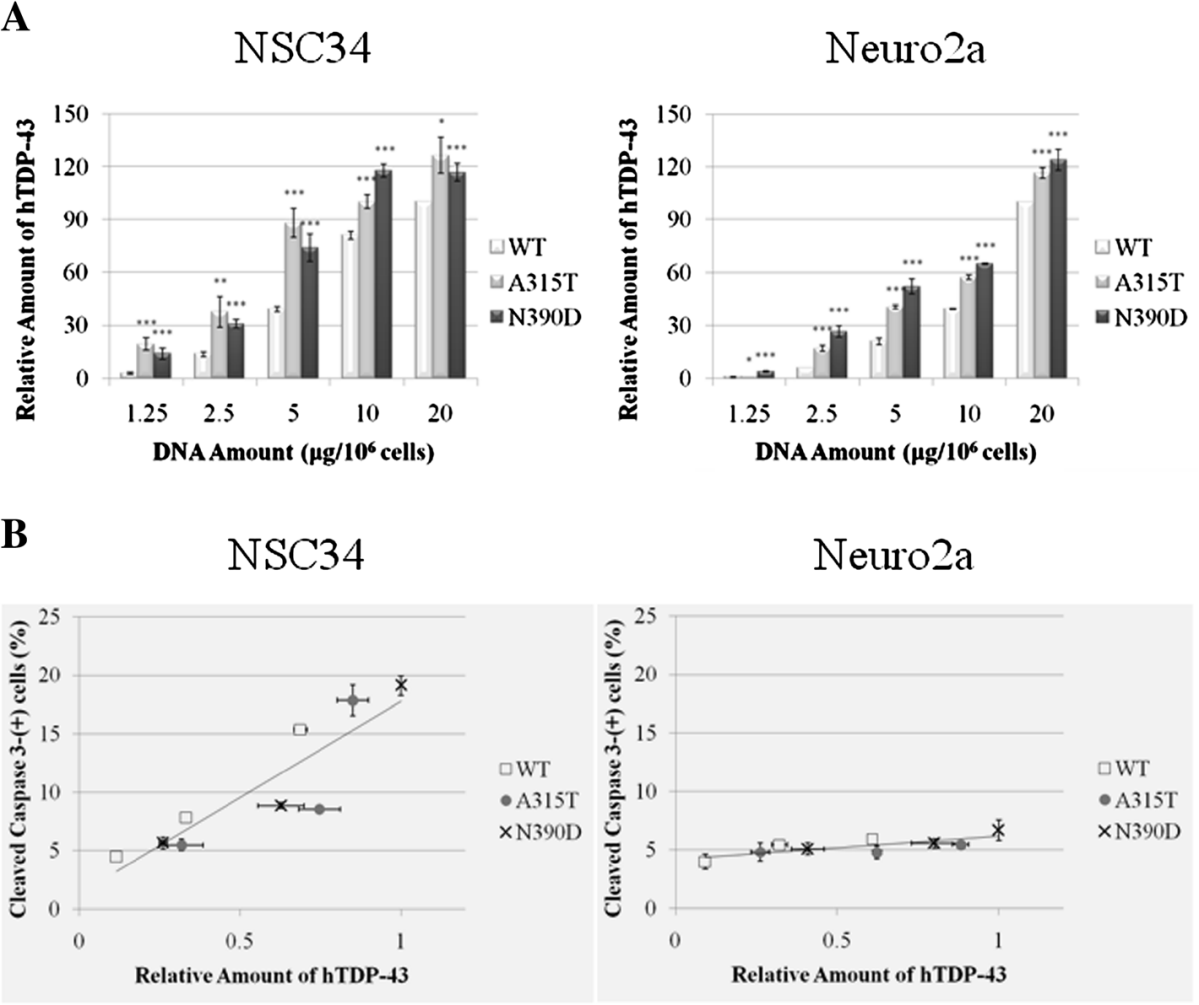
**hTDP-43 protein dose-dependent apoptotic death of NSC34 cells. (A)** Higher levels of MT hTDP-43 than WT hTDP-43 at the same dose(s) of DNA transfection. NSC34 and Neuro2a cells were transfected with different doses (μg/ 10^6^ cells) of the expression plasmids. At 72 hr post-transfection, the levels of the exogenous hTDP-43 expressed were compared by Western blotting using the anti-hTDP-43 antibody 2E2D3, with the mouse Hsp70 level as the internal control. The means of the relative levels of hTDP-43 obtained from three independent experiments (S.D.) are then plotted in the 2 panels, with the level of the WT hTDP-43 in cells with the transfection dose of 20 μg DNA/ 10^6^ cells as 100. Note the higher levels of the MT hTDP-43 than WT hTDP-43 in both NSC34 and Neuro2a cells at all doses of transfection. *, *p<*0.05; **, *p<*0.01; ***, *p<*0.001. **(B)** The % of transfected cells that were cleaved caspase 3-positive as a function of the relative amounts of hTDP-43. The plots were derived from the combined data of Figures 2 and 3A. The amounts of hTDP-43 in NSC34 cells or Neuro2a cells transfected with 20 μg of pEF-hTDP-43^N390D^ are assigned the value of 1.

The relatively higher death incidence of NSC34 cells as caused by MT hTDP-43 than the WT hTDP-43 (Figure 2B) could be due to that ALS-associated TDP-43 mutations affecting certain motor neuronal cell-specific functions of hTDP-43 or cellular pathways. Alternatively, these mutations might exert their effects by elevating the steady-state level of the MT hTDP-43 through stabilization of the protein. Significantly, when the total cellular extracts of transfected cells were analyzed by Western blotting, the steady-state levels of the two MT hTDP-43 were indeed higher than the WT TDP-43, and this was the case over a range of the amounts of plasmid DNA used for transfection (left panel of Figure 3A and Table 1). Similar pattern was observed when the extracts of transfected Neuro2a cells were analyzed (right panel of Figure 3A and Table 1). In parallel to Additional file 2: Figure S2, the dose dependence of the steady state level of WT mTDP-43 in transfected NSC34 or Neuro2a cells was similar to WT hTDP-43 (Additional file 3: Figure S3).

**Table 1:** Levels of exogenous hTDP-43 proteins relative to the endogenous mTDP-43 in transfected cells*

	**Plasmid DNA amount (μg) used for transfection of 10**^**6 **^**cells**				
	**1.25**	**2.5**	**5**	**10**	**20**
**NSC34**					
**WT**	**0.26**	**0.56**	**0.73**	**0.87**	**1.1**
**A315T**	**0.54**	**0.77**	**0.80**	**1.0**	**1.3**
**N390D**	**0.61**	**0.86**	**0.93**	**1.3**	**1.5**
**Neuro2a**					
**WT**	**0.29**	**0.49**	**0.86**	**1.4**	**1.7**
**A315T**	**0.44**	**0.65**	**1.1**	**1.6**	**1.9**
**N390D**	**0.50**	**0.89**	**1.2**	**1.6**	**2.2**

* The levels of the exogenous hTDP-43 proteins relative to the endogenous mTDP-43 were measured first by Western blot analysis using the Gene Tex antibody recognizing both hTDP-43 and mTDP-43. The ratio of the intensity of the exogenous hTDP-43 to that of the endogenous mTDP-43 at each dose of the transfection was estimated and then calibrated against the average transfection efficiencies of the cells (50% for NSC34 and 70% for Neuro2a cells).

Since both the cytotoxicity of NSC34 cells caused by MT hTDP-43 (Figure 2B) and their steady-state levels (Figure 3A) were higher than WT hTDP-43 at the same dose(s) of DNA transfection, we speculated that the differences of cytotoxicities as caused by the MT and WT hTDP-43 might reflect mainly the differences of the steady-state levels of the proteins, instead of the sequence differences of the polypeptide per se. Indeed, as seen in the left panel of Figure 3B, the NSC34 cell death caused by the exogenous hTDP-43 proteins increased as a function of the relative amounts of the proteins, irrespective of whether the protein was the WT or the MT forms. As expected from the data of Figure 2B, the relatively lower cytotoxicity of the Neuro2a cells caused by the exogenous hTDP-43 remained similar over a range of the amounts of hTDP-43, WT or MT, expressed in the cells (right panel, Figure 3B).

**Figure 4:**
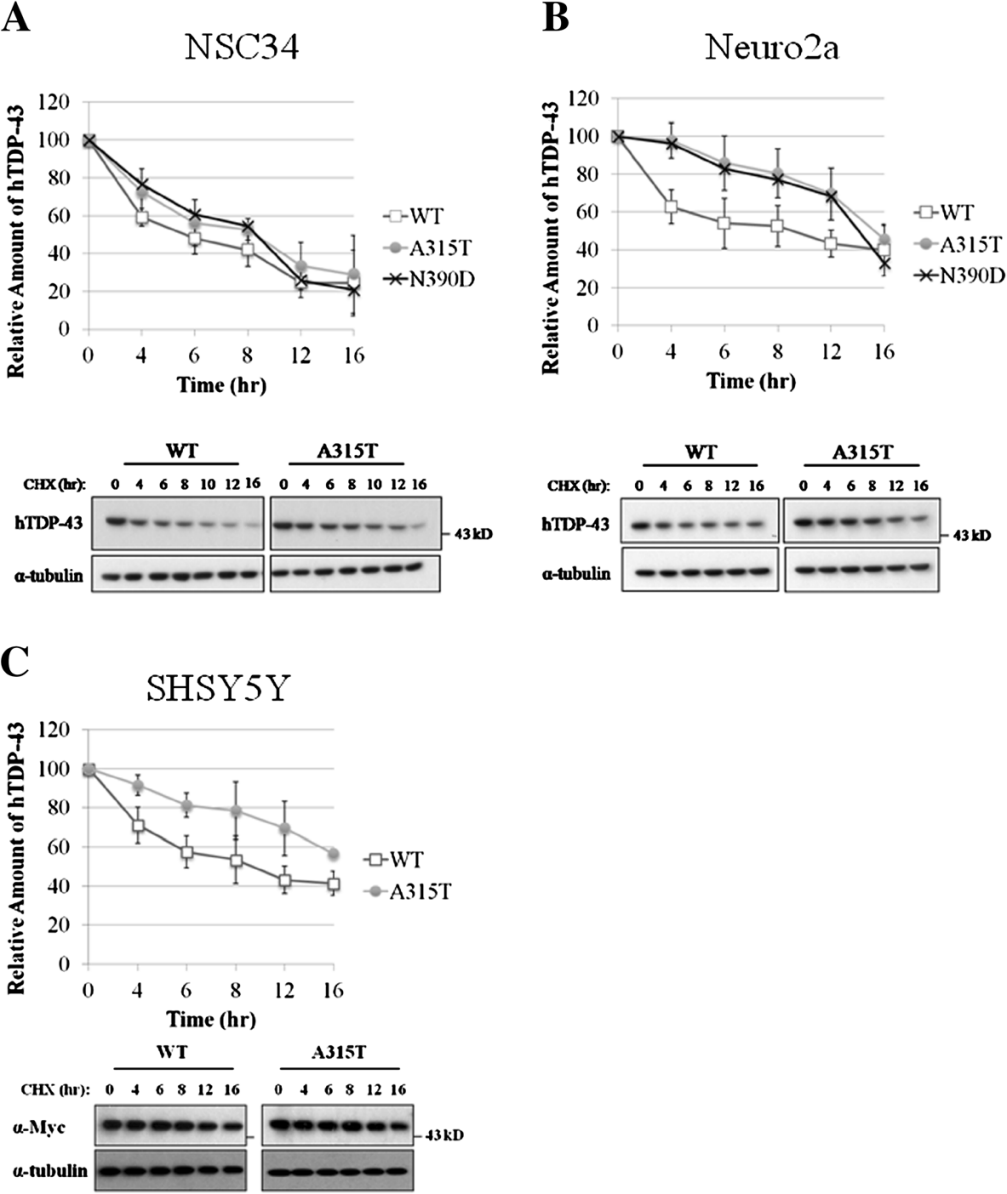
**Increased stabilities of hTDP-43 polypeptides with ALS-associated mutations.** The stabilities of the exogenous hTDP-43 in transfected NSC34 **(A),** Neuro2a **(B)**, and SHSY5Y **(C)** cells were measured by cycloheximide chase experiments as described in Methods. The Western blots of the exogenous WT hTDP-43 and MT hTDP-43^A315T^ in transfected cells are shown as the examples of analysis. The relative amounts of hTDP-43 at different hr of cycloheximide treatment are plotted in the upper two panels. The mean values of 3 different sets of the chase experiments are used for the plots. *p<*0. in NSC34, Neuro2a, and SHSY5Y cells.

We then examined whether the ALS-associated mutations stabilized hTDP-43 in NSC34, Neuro2a, and SHSY5Y cells. For this, we carried out cycloheximide chase experiments [36] (also see the experimental details in Methods) in these three types of cells. 10^6^ of the cells were transfected with 2.5 μg of the individual expression plasmids and then split into different aliquots at 20 hr post-transfection. These aliquots of cells were allowed to adhere for another 20 hr and then each aliquot was treated with cycloheximide for a different period of time. As exemplified in the Western blots in Figure 4, the amounts of the WT hTDP-43 in different types of the transfected cells, i.e. NSC34 (Figure 4A), Neuro2a (Figure 4B), SHSY5Y (Figure 4C), and HEK293T (data not shown) were significantly lower than those of the two MT hTDP-43 forms at each of the time points of cycloheximide treatment. This corresponded to increases of the half-life of hTDP-43, as the result of the ALS mutations, by approximately 2 to 4 hrs (Figure 4A), 6 to 8 hrs (Figure 4B), and 6 to 8 hrs (Figure 4C), respectively. The stabilization of hTDP-43 by the ALS mutations was in consistency with the higher steady-state levels of the MT hTDP-43 proteins than the WT hTDP-43 in transfected NSC34 cells (Figure 3).

### Both WT and MT hTDP-43 induced motor neuron cell death in a dose- dependent manner

The cell type-independent stabilization of hTDP-43 by A315T and N390D, as shown above, might be a general effect of most of the ALS-associated TDP-43 mutations. 3 others (Q298S, Q331K, and M337V) have been shown to stabilize hTDP-43 in HeLa cells and in primary fibroblast culture from a human patient [37]. Also, 5 other ALS-associated mutations (G298S, Q343R, G348C, N352S, and A382T) increased the protein half-life of hTDP-43 in Neuro2a cells [38]. In the latter study, it was also found that stabilization of hTDP-43 by the mutations was correlated with an early disease onset, but not related to the detergent insolubility and subcellular localization of hTDP-43 [38] This effects provides a reasonable explanation for the appearing-to-be higher motor neuron cyto-toxicity of the MT hTDP-43 forms than the WT when the cell death data from DNA transfection experiment(s) using the same amount(s) of the expression plasmids are compared (Figure 2B). In other words, overexpression of hTDP-43 is sufficient to cause dose-dependent apoptotic deaths of the motor neuronal NSC34 cells, irrespective of whether the overexpressed hTDP-43 is WT or carrying ALS-associated mutations (Figure 2B and 3B). With respect to the dose dependence, increase of the cellular level of the TDP-43 protein, with exogenous expression of either WT or MT forms of hTDP-43, by 50-200% (Table 1) would induce 5-20% of the transfected NSC34 cells to undergo apoptotic death (Figure 2B).

In interesting parallel with the dose-dependence of the cytotoxicity of hTDP-43 as derived from this study, previous transgenic mice [39-44] and transgenic *Drosophila*[45-47] experiments have suggested that elevation of the level of TDP-43, whether mutant forms or the wild type, is sufficient to cause TDP-43 proteinopathies. Also, overexpression TDP-43 in cultured human [48] and mouse cells [24,49] induced cytotoxicity. These transgenic cell culture and animal studies are in interesting correlation with the finding of elevated levels of hTDP-43 expression in some cases of ALS and FTLD-U [50,51]. Thus, the steady-state level of TDP-43 could be one determining factor for the occurrence and/ or progression of neurodegeneration in TDP-43 proteinopathies. Finally, since the stabilization of hTDP-43 by the ALS-associated mutations occurs in all the cell types that we have tested, it is likely that motor neuronal cells have a relatively low tolerance to the elevated amount of hTDP-43 when compared to other types of cells.

Take together all of the above, we suggest that pathogenesis of ALS could be due to the selective neurotoxicity of the spinal motor neurons caused by elevated level of hTDP-43, which in turn results from mis-regulation of the hTDP-43 metabolism due to different ALS-associated gene mutations including those within the *TARDBP* gene itself. How the ALS-associated mutations in hTDP-43 stabilize the protein and why the spinal motor neurons have a relatively low tolerance to the elevated level of TDP-43 remain to be investigated.

## Conclusions

In conclusion, based on our data and previous transgenic TDP-43 studies in animals or cell cultures, we suggest that one major common consequence of the different ALS-associated TDP-43 mutations is the stabilization of the hTDP-43 polypeptide. The resulting elevation of the steady state level of hTDP-43 in combination with the relatively low tolerance of the spinal motor neurons to the increased amount of hTDP-43 lead to the neurodegeneration and pathogenesis of ALS, and of diseases with TDP-43 proteinopathies in general.

## Competing interests

The authors declare that they have no competing interests.

## Authors’ contributions

LSW carried out the experiment work and analyzed data. LSW, WCC and CKJS designed the study, coordinated the experiments and analyzed data. LSW and CKJS wrote the manuscript. All authors read and approved the final manuscript.

## Supplementary Material

Additional file 1: Figure S1 Comparison of apoptotic deaths of cells with exogenous expression of WT hTDP-43 and mTDP-43. Apoptotic cell death was assayed by the activities of caspase 3/ 7 at 24 hr and 72 hr post-transfection of NSC34 and Neuro2a cells with plasmids expressing hTDP-43 and mTDP-43, respectively (5 ug/ 10^6^ cells), as described in the legend of Figure 2A. Mock, cells without transfection; C, cells transfected with the pEF vector; Stau. 5 uM, cells treated with 5 uM of staurosporine for 6 hr to induce apoptosis. The folds of the caspase activities relative to that of the Mock sample were calculated and shown. Note the lack of effect on the caspase 3/ 7 activities by the exogenously expressed hTDP-43 or mTDP-43. The differences in the caspase 3/ 7 activities among the variants were assessed by the ANOVA test. Click here for file

Additional file 2: Figure S2 Comparison of the plasmid dose-dependent apoptotic deaths induced by exogenous hTDP-43 and mTDP-43. Apoptotic deaths of transfected NSC34 cells and Neuro2a cells at 72 hr post-transfection with different amounts of the expression plasmids were assayed by immunofluorescence staining with the antibodies anti-Myc and Ac-cap 3, as described in the legend of Figure 2B. Means of three independent experiments (S.D.) are plotted in the upper 2 panels, with the % of hTDP-43-positive cells that are also Ac-cap 3-positive as a function of the doses of transfection. Approximately 1% of cells transfected with the pEF vector were Ac-cap3 positive (* on the y axes of the two plots). Representative photographs are shown below the plots. Scale bar, 10 μm. The differences in% of the Ac-cap 3-positive cells among the variants were assessed by the ANOVA test. Click here for file

Additional file 3: Figure S3 Expression plasmid dose-dependent increase of hTDP-43 and mTDP-43 in trasnfected NSC34 and Neuro2a cells. NSC34 and Neuro2a cells were transfected with different doses (μg/ 10^6^ cells) of the appropriate expression plasmids. At 72 hr post-transfection, the levels of the exogenous hTDP-43 and mTDP-43 proteins were compared by Western blotting with use of anti-Myc. The mouse Hsp70 and tubulin were analyzed as the internal control. The means of the relative levels obtained from three independent experiments (S.D.) are plotted in the lower 2 panels, with the level of the exogenous hTDP-43 in cells with the transfection dose of 20 μg plasmid DNA/ 10^6^ cells as 100. The differences in the relative levels of the Myc-tagged hTDP-43 or mTDP-43 among the variants were assessed by the ANOVA test. Note the similar levels of hTDP-43-Myc and mTDP-43-Myc at each dose of the expression plasmid(s) used. Click here for file
