# Supplementary figures and images for: Similar dose-dependence of motor neuron cell death caused by wild type human TDP-43 and mutants with ALS-associated amino acid substitutions
Source: J Biomed Sci. 2013 May 30;20(1):33. doi: 10.1186/1423-0127-20-33 (PMC3684520; doi:10.1186/1423-0127-20-33)

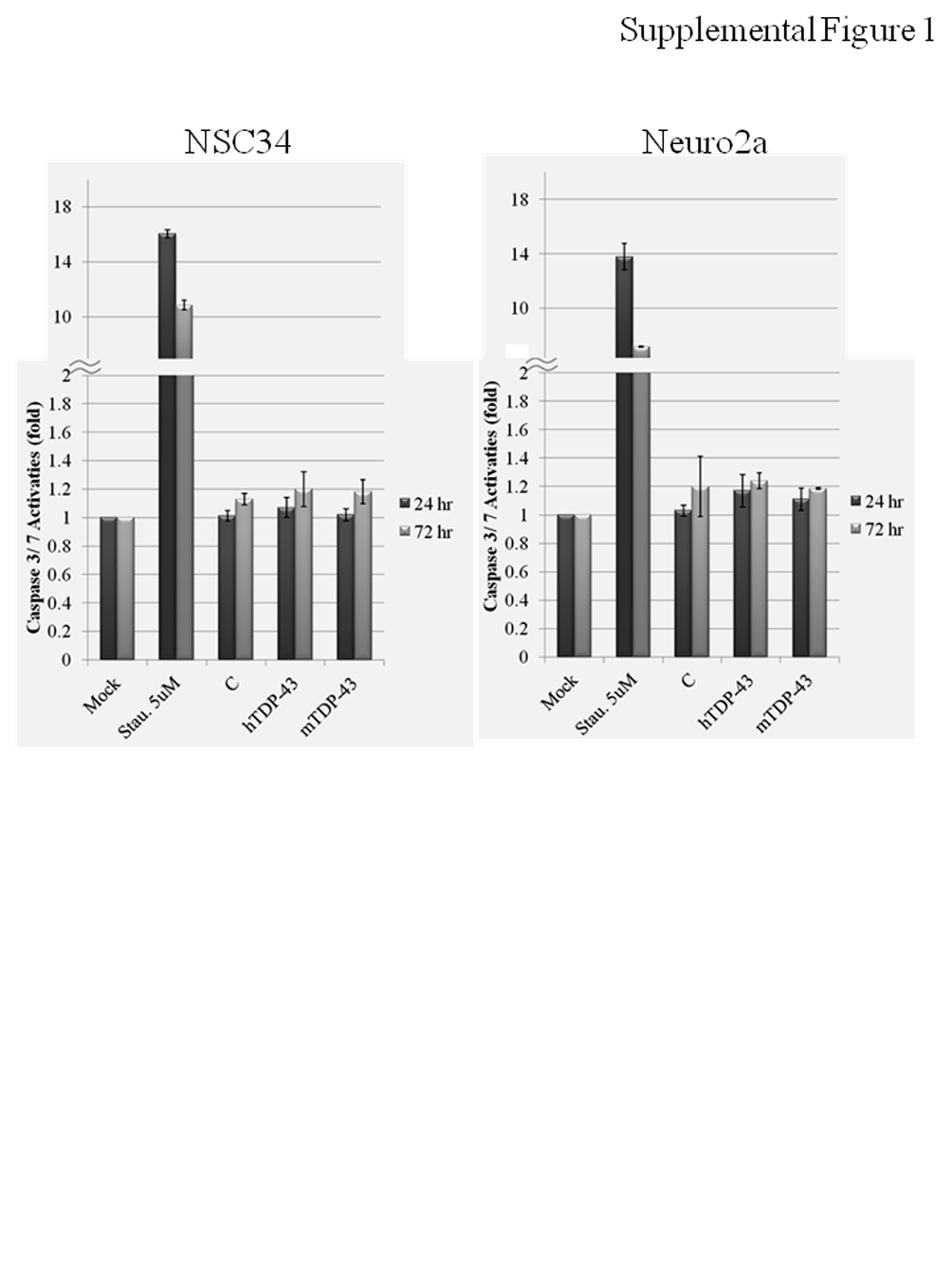

Supplement: Additional file 1: Figure S1 — Comparison of apoptotic deaths of cells with exogenous expression of WT hTDP-43 and mTDP-43. Apoptotic cell death was assayed by the activities of caspase 3/ 7 at 24 hr and 72 hr post-transfection of NSC34 and Neuro2a cells with plasmids expressing hTDP-43 and mTDP-43, respectively (5 ug/ 106 cells), as described in the legend of Figure 2A. Mock, cells without transfection; C, cells transfected with the pEF vector; Stau. 5 uM, cells treated with 5 uM of staurosporine for 6 hr to induce apoptosis. The folds of the caspase activities relative to that of the Mock sample were calculated and shown. Note the lack of effect on the caspase 3/ 7 activities by the exogenously expressed hTDP-43 or mTDP-43. The differences in the caspase 3/ 7 activities among the variants were assessed by the ANOVA test. [file 1423-0127-20-33-S1.tiff]

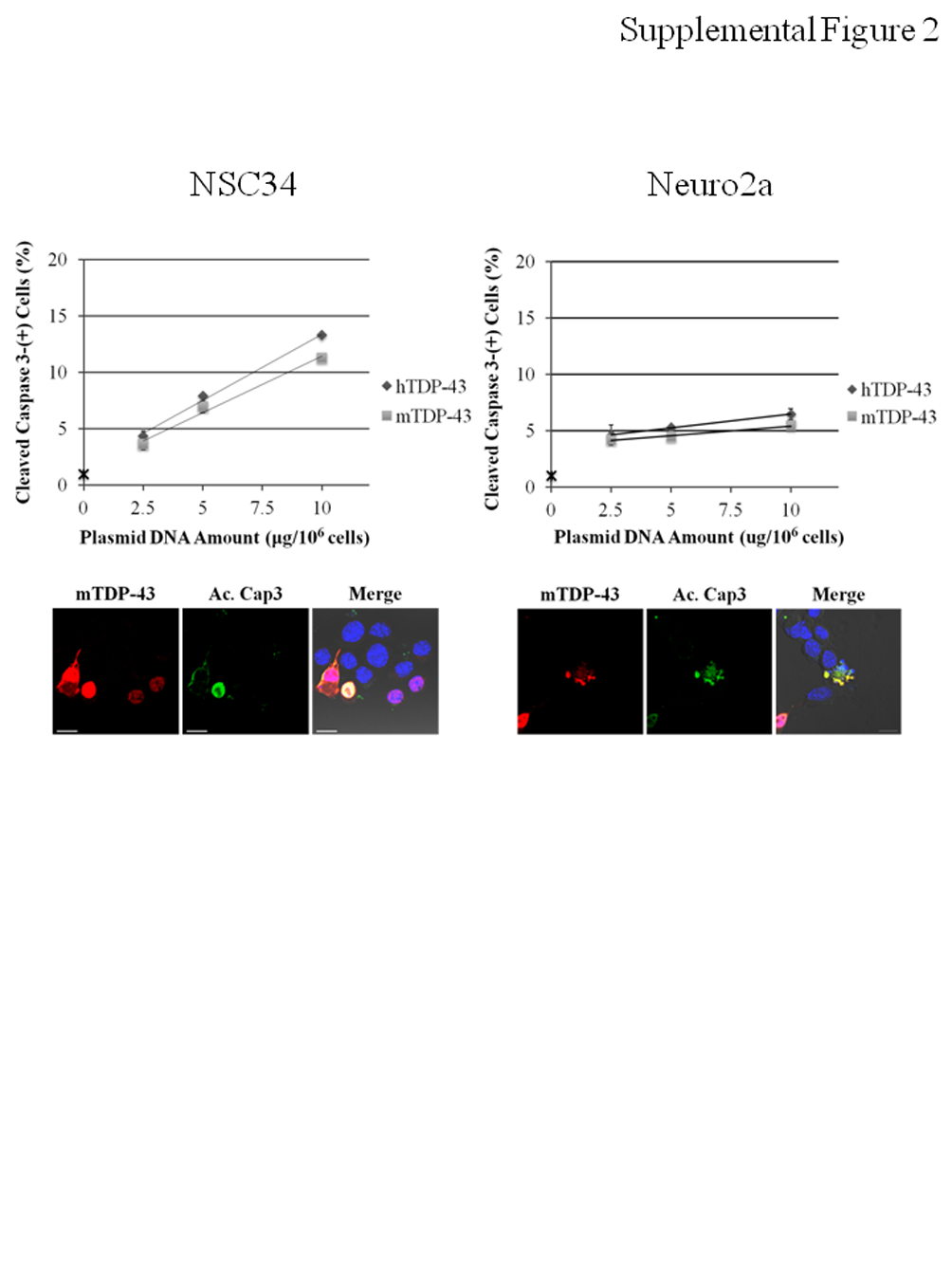

Supplement: Additional file 2: Figure S2 — Comparison of the plasmid dose-dependent apoptotic deaths induced by exogenous hTDP-43 and mTDP-43. Apoptotic deaths of transfected NSC34 cells and Neuro2a cells at 72 hr post-transfection with different amounts of the expression plasmids were assayed by immunofluorescence staining with the antibodies anti-Myc and Ac-cap 3, as described in the legend of Figure 2B. Means of three independent experiments (S.D.) are plotted in the upper 2 panels, with the % of hTDP-43-positive cells that are also Ac-cap 3-positive as a function of the doses of transfection. Approximately 1% of cells transfected with the pEF vector were Ac-cap3 positive (* on the y axes of the two plots). Representative photographs are shown below the plots. Scale bar, 10 μm. The differences in% of the Ac-cap 3-positive cells among the variants were assessed by the ANOVA test. [file 1423-0127-20-33-S2.tiff]

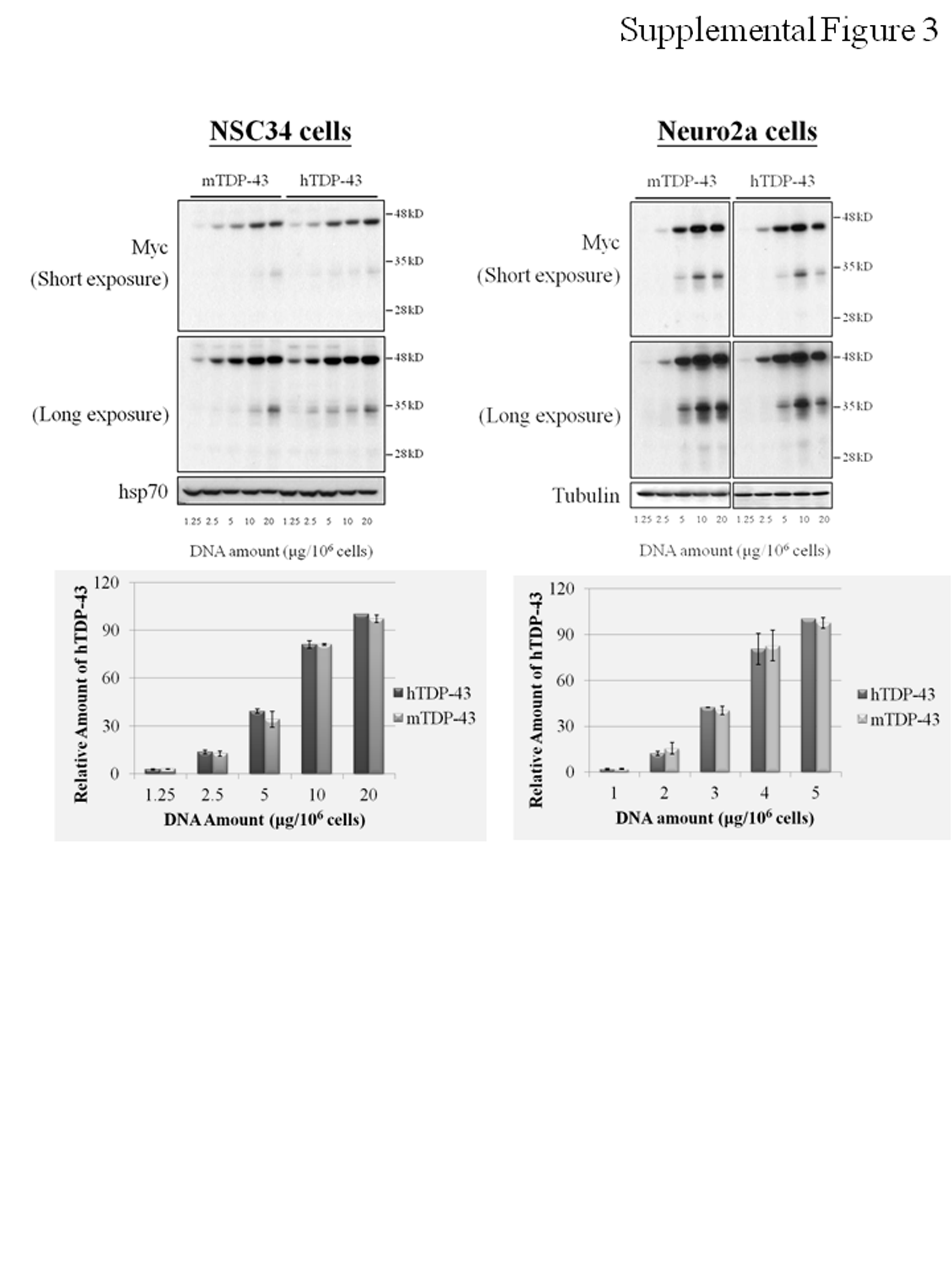

Supplement: Additional file 3: Figure S3 — Expression plasmid dose-dependent increase of hTDP-43 and mTDP-43 in trasnfected NSC34 and Neuro2a cells. NSC34 and Neuro2a cells were transfected with different doses (μg/ 106 cells) of the appropriate expression plasmids. At 72 hr post-transfection, the levels of the exogenous hTDP-43 and mTDP-43 proteins were compared by Western blotting with use of anti-Myc. The mouse Hsp70 and tubulin were analyzed as the internal control. The means of the relative levels obtained from three independent experiments (S.D.) are plotted in the lower 2 panels, with the level of the exogenous hTDP-43 in cells with the transfection dose of 20 μg plasmid DNA/ 106 cells as 100. The differences in the relative levels of the Myc-tagged hTDP-43 or mTDP-43 among the variants were assessed by the ANOVA test. Note the similar levels of hTDP-43-Myc and mTDP-43-Myc at each dose of the expression plasmid(s) used. [file 1423-0127-20-33-S3.tiff]
